# Between Borders and Bodies: Rethinking National and Human Security

**DOI:** 10.1177/00207020261452704

**Published:** 2026-06-19

**Authors:** Luana Figueiredo, Makeda Smith

**Affiliations:** 33530 University of Toronto, Toronto, Canada

**Keywords:** Canada, national security, sovereignty, total defence, NATO

## Abstract

This conversation piece examines how Canada navigates the challenges of national and human security. Drawing on insights from experts in defence studies, foreign policy, international law, and human rights, it explores the financial and institutional costs of sovereignty, the fragility of multilateral alliances, and the prospect of American ambivalence or aggression. Shifts toward ad hoc coalitions, total defence models, and domestic resilience are highlighted, while showing how border regimes, securitization practices, and selective legal enforcement reproduce racialized violence and erode humanitarian norms. The article argues that Canada's security cannot be outsourced or pursued solely through military preparedness; it must be grounded in justice, equity, and the primacy of human rights. Protecting the state, it contends, is meaningless if people within its borders are not safe and free, and security policy must therefore integrate national defence with robust commitments to human security.

In an increasingly volatile geopolitical landscape, Canada finds itself navigating the complex and often competing demands of national security and human security. As threats to its sovereignty and territory mount, the country faces urgent questions about how to protect both the state and the people within it. This conversation piece explores the pressing challenges decision-makers must confront: How can Canada defend its sovereignty without sacrificing human rights? Is it possible to enhance national defence without deepening systemic injustices? What are the challenges of building a whole-of-society or total defence model in Canada? Here's what leading experts in the field say every Canadian should know about the future of national security and human security.

## National security

*Dr. Barbara J. Falk, Professor, Department of Defence Studies at the Canadian Forces College* ^1^

Canada has not yet confronted the full financial, political, and institutional costs of sovereignty. Germany's post-reunification solidarity tax is a reminder that significant state challenges and expenditures require tangible financial resources, not simply rhetorical commitment. Canada could adopt a similar approach to avoid health care and education vs. defence trade-offs, either in terms of perception or budgetary reality. Canadians must reconsider what they expect of national defence as the country faces mounting pressures: protecting the Arctic, countering foreign interference, and navigating the instability of US political discourse. Security cannot be outsourced – it depends on a whole-of-society approach that embeds resilience in civic institutions, infrastructure, and public consciousness. Sovereignty is not a one-time achievement; it must be reinforced daily through education, policy, and sustained public investment. It is neither automatic nor guaranteed by allies – it rests on national will and institutional readiness.

*Dr. Stéfanie von Hlatky, Professor, Department of Political Studies at Queen's University* ^2^

The US ambivalence toward NATO threatens to stall or dismantle progress on critical international priorities, with climate security and Women, Peace, and Security being the most critically affected. While NATO's institutional structures have endured for now, we must question how political contestation will affect them over the course of President Trump's term. Allies have already hedged their bets, building internal capacity and prioritizing military readiness, boosting public engagement on defence issues, ensuring robust critical infrastructure, and other developments that have already taken hold in NATO countries with renewed total defence force models. Security in the contemporary context cannot be approached reactively; domestic resilience must be recognized as a strategic asset for building credible collective defence and deterrence. For Canada, this means enhancing its international voice and upholding its values in a way that is strongly anchored in sustained defence investments and heightened national preparedness.

*Dr. Veronica Kitchen, Associate Professor, Department of Political Science at the University of Waterloo* ^3^

Experts and policymakers both recognize the need for Canada to have updated, agile security structures capable of responding to Canada-specific risks. Canada needs to build more independence into its intelligence institutions, to build more capacity to address domestic crise s into the Canadian Armed Forces, and to address internal structural limitations, such as the RCMP's dual role in law enforcement and national security. It will not be possible to do everything that may be desirable all at once, and it is necessary for us to have a national conversation about what our priorities should be and how we will pay for them. This is particularly important in an era where American security priorities are shifting, often away from threats like right-wing extremism and gender-based violence that Canada may wish to continue to prioritize. The real question is not whether we are over-reliant on the United States or whether our partners can protect us, but whether we maintain the capacity and political space to enact policies that reflect interests and values defined in Canada.

*Dr. Marion Laurence, Assistant Professor, Department of Political Science at Dalhousie University* ^4^

Canada's traditional foreign policy tools and strategies, especially those that depend on multilateral cooperation, are becoming increasingly unstable. There is a turn away from formal institutions and toward “ad hocism,” where temporary, interest-based coalitions are formed to manage emerging threats and geopolitical crises. This shift exposes Canada to a new kind of strategic volatility: Canada can no longer count on NATO's cohesion or US leadership to hold. Strategic resilience now means investing in adaptability, recalibrating foreign policy assumptions, and preparing for a more fragmented world order. Nostalgia for past alliances risks obscuring present realities; adherence to outdated models may pose a greater danger than the threats themselves. Confronting the geopolitical landscape as it is, rather than as it once was, is essential to ensuring long-term security and relevance on the global stage.

*Dr. Aisha Ahmad, Associate Professor, Department of Political Science at the University of Toronto Scarborough* ^5^

In this hostile new world order, Canada must prepare for the once-unthinkable scenario of American military aggression. Not only has the Canada-US relationship deteriorated, but there is now a very real possibility that future threats to Canadian security and sovereignty will come from the Americans themselves. It is therefore imperative that Ottawa adopt a new approach to national defence. However, given its severe power asymmetry with the US, there are only three realistic options on the table: shoring up other global alliances, adopting a total defense model akin to the Scandinavians, or daring to acquire nuclear weapons. None of these options is as comfortable or secure as the bygone “rules-based international order”, but that era is dead. There is no magic deterrence fairy that will save Canadians from superpower aggression, so Ottawa must take bold action to address this tough new reality.

## Human security

*Dr. Jennifer Welsh, Professor, Department of Political Science at McGill University* ^6^

We are in a time where international humanitarian law has eroded. Although these legal frameworks were originally built on reciprocity and mutual interest, they have weakened over time, increasingly placing national interests above the protection of civilians. In moments of conflict, narratives of victory tend to dominate, pushing human suffering and civilian lives to the margins. This shift reinforces a growing divide between the powerful and the vulnerable, where human rights are no longer treated as universal but as conditional, depending on geopolitical influence. National security concerns have overtaken the original goal of minimizing harm and protecting people. To rebuild trust and effectiveness, there is a need for local and grassroots efforts rather than top-down international solutions.

*Dr. Nicole Bernhardt, Assistant Professor, Department of Political Science at the University of Toronto Scarborough* ^7^

In a world marked by rising cross-border tensions, racial justice and human rights are increasingly under threat. This current era of hostility is marked by states turning towards the same divisive practices of surveillance and securitization that have consistently perpetuated harm against racialized and marginalized groups. Meaningful cross-border connections and grassroots movements are needed to protect the primacy of human rights, placing them at the center of all governance considerations. When national security strategies focus on securing borders above fundamental rights, we lose a crucial sense of community, togetherness, and social justice. This has dire consequences for human rights because when national interests become more important than human dignity, we lose the very foundation of human rights. We need movements, not just institutions, to hold the line.

*Dr. Martha Balaguera, Assistant Professor, Department of Political Science at the University of Toronto Mississauga* ^8^

State-sanctioned violence and aggressive border control are not new Trump-era politics but are, in fact, deeply rooted in long-standing legal and political structures. These patterns persist across governments, revealing fundamental flaws in systems that claim to uphold justice. Individuals labeled as “threats” are routinely denied due process and treated as security risks rather than as people with rights. While legal frameworks are necessary, they often fail when weaponized against marginalized communities, particularly racialized asylum seekers such as Haitians, Central Americans, Mexicans, and, more recently, Venezuelans. The border functions not only as a physical divide but also as a legal barrier, where human rights collapse under the weight of rigid categorization. Balaguera's analysis calls for a reimagining of the law that actively works to protect dignity, equity, and human security.

*Dr. Irma Spahiu, Assistant Professor, Department of Political Science at the University of Toronto Scarborough* ^9^

Legal tools such as Canada's Charter of Rights and Freedoms and the Oakes Test are only meaningful when applied fairly and intentionally. Too often, these frameworks are used selectively or symbolically, failing to protect those most at risk. We must question not only the content of the law, but also how and when it is applied. We must be cautious against equating national security with public safety, especially when that narrative justifies the erosion of rights. Legal violence often hides behind claims of protection. For law to serve human security, it must be rooted in justice, not fear. It is imperative that we challenge dominant security narratives and ensure that legal systems protect people rather than threaten them.

*Professor Ghizal Haress, Adjunct Professor at the University of Toronto* ^10^

Shifting our attention toward Afghanistan, Canada's role in protecting human rights reveals how its military, diplomatic, and financial involvement helped build fragile institutions that eventually collapsed. While Canada contributed to institution-building, its withdrawal was hasty and excluded meaningful participation in peace negotiations to safeguard the rights of Afghans, particularly women. Abandoning Afghanistan to crisis also carries hard security repercussions: a collapsed state can become a breeding ground for transnational threats, from extremism to narcotics trafficking, that ultimately affect global and Canadian security. Canada's legacy demands continued engagement through humanitarian assistance, refugee support, political pressure, and advocacy for human rights and the rule of law, including ensuring Afghan women are meaningfully included in the case Canada has brought against the Taliban at the International Court of Justice, so their voices, experiences, and perspectives are reflected in the process.

## Navigating the new threat landscape

As Canada navigates a volatile new international order, it must balance its national security concerns with its firm commitment to upholding human rights and the rule of law. This challenge speaks to a critical question: what are we protecting, and at what cost? Amid rising geopolitical tensions and real threats to Canadian sovereignty and security, enhancing national defence has rightly become a key government priority. However, as the experts in this Conversation piece explain, this imperative cannot come at the expense of people's rights, dignity, and safety.

As the contributors emphasized, protecting the state means very little if the people within it are not also safe and free. The real challenge is therefore not choosing between national and human security but finding a way to integrate both objectives. Security is not measured solely by military strength or alliances, but also by justice, equity, and inclusion. Canada's security thus depends on how well it defends human rights, not just on how prepared the country is to respond to threats.

## Notes

1. Barbara Falk and Sara Skinner, *The Responsibility to Protect: A Normative Shift from Words to Action? International Peacekeeping* 23, no. 1 (2016): 1–13.

2. Joakim Berndtsson et al., eds., *Total Defence Forces in the Twenty-First Century*, Human Dimensions in Foreign Policy, Military Studies, and Security Studies, no. 20 (McGill-Queen's University Press, 2023).

3. Veronica Kitchen, “Multilayered but Not Coordinated: National Security Policing in Canada after 9/11,” in Andrea Charron et al., eds., *The Legacy of 9/11: Views from North America* (McGill-Queen's University Press, 2023).

4. Caroline Dunton, Marion Laurence, and Gino Vlavonou, “Pragmatic Peacekeeping in a Multipolar Era: Liberal Norms, Practices and the Future of UN Peace Operations,” *Journal of Intervention & Statebuilding* 17, no. 3 (2023).

5. Aisha Ahmad, ed., *Securing Canada's Future: Vital Insights from Women Experts* (University of Toronto Press, 2024).

6. Jennifer Welsh and Marie-Joëlle Zahar, “What Future for Peace Operations?,” *Ethics & International Affairs* 38, no. 4 (2024): 404–417.

7. Nicole Bernhardt, “Refusing Human Rights Police Partnerships,” *Journal of Canadian Studies* 58, no. 3 (2025): 624–649.

8. Martha Balaguera et al., “Racial Frontiers: Hemispheric Logics of Haitians’ Displacement and Asylum in the Americas,” *Antipode* 56, no. 2 (2024): 379–399.

9. Irma Spahiu, “Courts: An Effective Venue to Promote Government Transparency? The Case of the Court of Justice of the European Union,” *Utrecht Journal of International and European Law* 31, no. 80 (2015).

10. International Human Rights Program, https://ihrp.law.utoronto.ca/page/overview/current-projects.

